# Addiction in Iran: The Need for Culturally and Religiously Adapted Preventive/ Recovery Programs

**DOI:** 10.5812/ijhrba.8003

**Published:** 2013-06-26

**Authors:** Sina Hafizi

**Affiliations:** 1 School of Medicine, Tehran University of Medical Sciences, Tehran, IR Iran

**Keywords:** Religion, Addiction, Spirituality

Dear Editor,

Iran as a developing country is experiencing major changes at different economic and cultural levels. Along with all these changes, the population is also growing and number of young people is increasing. The alarm bells are ringing as the new studies show the high prevalence of addiction and risky behaviors among Iranian youth population (1). Previously studies on addiction in Iran have reported the prevalence of opium addiction to be between 1.2 to 8.8% (2, 3). Addiction is a national issue and affecting a large number of people throughout the country and it is important to be addressed through national programs. Religion is a well-established source of comfort and relief through human history. Today, scientific studies have proved the major role that religious belief could play in mental and physical health (4). To this fact, religious based therapies and treatments approaches are now widely used in the health programs especially in psychiatry and psychology (5). Many of the current well known psychotherapies such as mindfulness, Alcoholic Anonymous (AA), and 12-steps programs are rooted in religious beliefs and concepts. Despite the growing interest toward the research about religion and health in the world (6), studies seeking this relationship in Iran are limited and lacking. Religion and religious practice could have both protective and healing effects. Evidences from studies conducted mostly in western countries have shown a significant fewer number of drug addicted persons among more religious people (7). On the other hand, religion can help recovery from addiction mostly through increasing social supports and enhancement of positive emotions. A study which has been conducted with a sample of drug users in Brazil, suggests that religion-based treatments could be used beside conventional addiction therapies as a complementary treatment method (8). Religious beliefs could promote pro social behavior and a sense of meaning in life. As Koenig (7), mentions in his article titled “Research on Religion, Spirituality, and Mental Health: A Review”, religion and religious beliefs could offer psychological and emotional support throughout hard times in life. Religion could provide free full-time mental health services to anyone in any place. In conclusion despite the significant role of religion in shaping both personal and public life of Iranians, there is not any religiously and culturally adapted preventive/recovery program available especially for addiction. This goal could be reached only by doing scientific research on how religion concepts should be used in making a comprehensive plan for prevention/recovery from addiction. A good study needs good measures. Although some limited numbers of measures have been validated according to the cultural and religious difference and are now available in Farsi (9), much more researches needs to be done (10).
